# The complete mitochondrial genome of the freshwater crab *Chinapotamon maolanense* (Decapoda: Brachyura: Potamoidea)

**DOI:** 10.1080/23802359.2020.1773330

**Published:** 2020-06-08

**Authors:** Ying-yi Cui, Lin-bo Shi, Wen-bin Ji, Shu-xin Xu, Chun-chao Zhu, Xian-min Zhou, Jie-xin Zou

**Affiliations:** a Research lab of Freshwater Crustacean Decapoda & Paragonimus, School of Basic Medical Sciences, Nanchang University, Nanchang City, Jiangxi Province, People’s Republic of China; b Key Laboratory of Poyang Lake Environment and Resource Utilization, Ministry of Education, Nanchang University, Nanchang City, Jiangxi Province, People’s Republic of China

**Keywords:** Brachyuran, complete mitochondrial genome, phylogenetic, *Chinapotamon maolanense*

## Abstract

The complete mitochondrial genome of *Chinapotamon maolanense* was obtained for the first time. The complete mitochondrial genome of *C. maolanense* is 17,130 bp in length, including 13 protein-coding genes, 22 tRNA genes, 2 rRNA genes, and 1 control region. In addition, the mitogenome has 18 noncoding regions ranging from 1 to 1553 bp in length.

The species *Chinapotamon maolanense* (Dai 1999) (Crustacea: Malacostraca:Decapoda: Brachyura: Potamidae: *Chinapotamon*) is distributed in the karst landform area of Guizhou Province, China (Jie-Xin et al.). The complete mitochondrial genome of *C. maolanense* was obtained for the first time.

An adult specimen of *C. maolanense* was collected from Panzhai Village, Dawn township aquatic animals, Libo County, Qiandongnan Miao and Dong Autonomous Prefectu, Guizhou Province, China in 2017 (N25.234444°, E 108. 029527°). The sample has been deposited in the Laboratory Specimen Library of Freshwater Crustacean Decapoda & Paragonimus, School of Basic Medical Sciences, Nanchang University, Nanchang, Jiangxi, PR China & National Parasite Germplasm Resources Specimen Library of China with a catalogue number of NCUMCP1960. The sample was stored in 95% ethanol prior to extraction at room temperature before sequence analyses. Genomic DNA extraction, sequencing, gene annotation, and phylogenetic analyses were performed according to the method described by Plazzi et al. (2013). The Bayesian Inference (BI) method was performed using MrBayes vers. 3.2 (Ronquist et al. 2012), with best model GTR + I + G selected using jModelTest vers. 2.1.7. The maximum-likelihood (ML) method was performed using MEGA 6 (Tamura et al. 2013).

*Chinapotamon maolanense* (GenBank accession no. MT134100) has a full-length mitochondrial genome of 17,130 bp and shares the same 37 genes (13 protein-coding genes, 22 tRNA genes, and 2 rRNA genes) and 1 control region (CR) as the typical metazoan mitochondrial genome (Yan et al.), with a high A + T bias (71.7%). The CR of *C. maolanense* is located in a typical crustacean position (between *12SrRNA* and *tRNA^Ile^*), it is 1553 bp in length, and has a higher A + T bias (76.5%) than does the mitochondrial genome. Among these genes, 23 are located in the H-strand, and 14 are located in the L-strand.

The mitochondrial genome of *C. maolanense* has 18 noncoding regions and is between 1–1553 bp in length, with the longest noncoding region between *12S rRNA* and *tRNA^Ile^* which is considered to be the CR; there are nine gene overlap areas, with a total length of 25 bp, and those areas are between 1 and 7 bp in length, with the longest gene overlap area between *ND4* and *ND4L*.

The mitochondrial genome of *C. maolanense* contains 13 protein-coding genes. Similar to other Brachyura mitochondrial genomes, 9 protein-coding genes are located in the H chain (*COX1*, *COX2*, *COX3*, *ATP6*, *ATP8*, *ND2*, *ND3*, *ND6*, and *CYTB*), and the remaining four are located in the L chain (*ND1*, *ND4*, *ND4L* and *ND5*). The initiator codon of protein-coding genes are ATG (*COX1*, *COX2*, *ATP8*, *COX3*, *ND4*, *ND4L*, *Cyt b*, *ND1* and *ND2*), and ATA (*ATP6*, *ND3*, *ND5* and *ND6*); the termination codon are TAA (*COX1*, *COX2*, *ATP6*, *COX3*, *ND4*, *ND4L*, *ND6*, *ND1* and *ND6*), and TAG (*ATP8*), and incomplete termination codon T (*ND3*, *ND5* and *Cyt b*). The A + T bias of the protein-coding gene is 69.0%.

The mitochondrial genome of *C. maolanense* possesses two rRNA genes, *16S rRNA* and *12S rRNA*, which are located in the same L chain as other Brachyura mitochondrial genomes. The length of the *16S rRNA* and *12S rRNA* genes is 1318 bp and 824 bp, respectively; *16S rRNA* is between *tRNA^Leu(CUN)^* and *tRNA^Val^*, while *12S rRNA* is between *tRNA^Gln^* and the CR.

*Chinapotamon maolanense* has 22 tRNAs in common, and most tRNAs have a typical clover structure, with the exception of *tRNA^Ser(AGN)^* which lacks the dihydrouracil (DHU) arm (Ohtsuki et al. 2002). The 22 tRNA genes are between 61 (*tRNA^Arg^*) and 74 bp (*tRNA^Val^*) in length. Additionally, there are certain base mismatches, including 36G–T mismatches, 3A–C mismatches, 1A–G mismatches, 1T–C mismatches, 1A–A mismatches, and 2T–T mismatches.

The phylogenetic position of *C.maolanense* in mitogenome relative to other freshwater crabs mitogenomes is determined by applying the BI and ML methods on 13 PCGs (Figure 1). Our results indicate that as one of the freshwater crabs, *C. maolanense* is the sister group with *Huananpotamon lichuanense* and *Sinolapotamon patellifer*. This result is consistent with morphological classification and other molecular analyses (Dai 1999).

**Figure 1.**
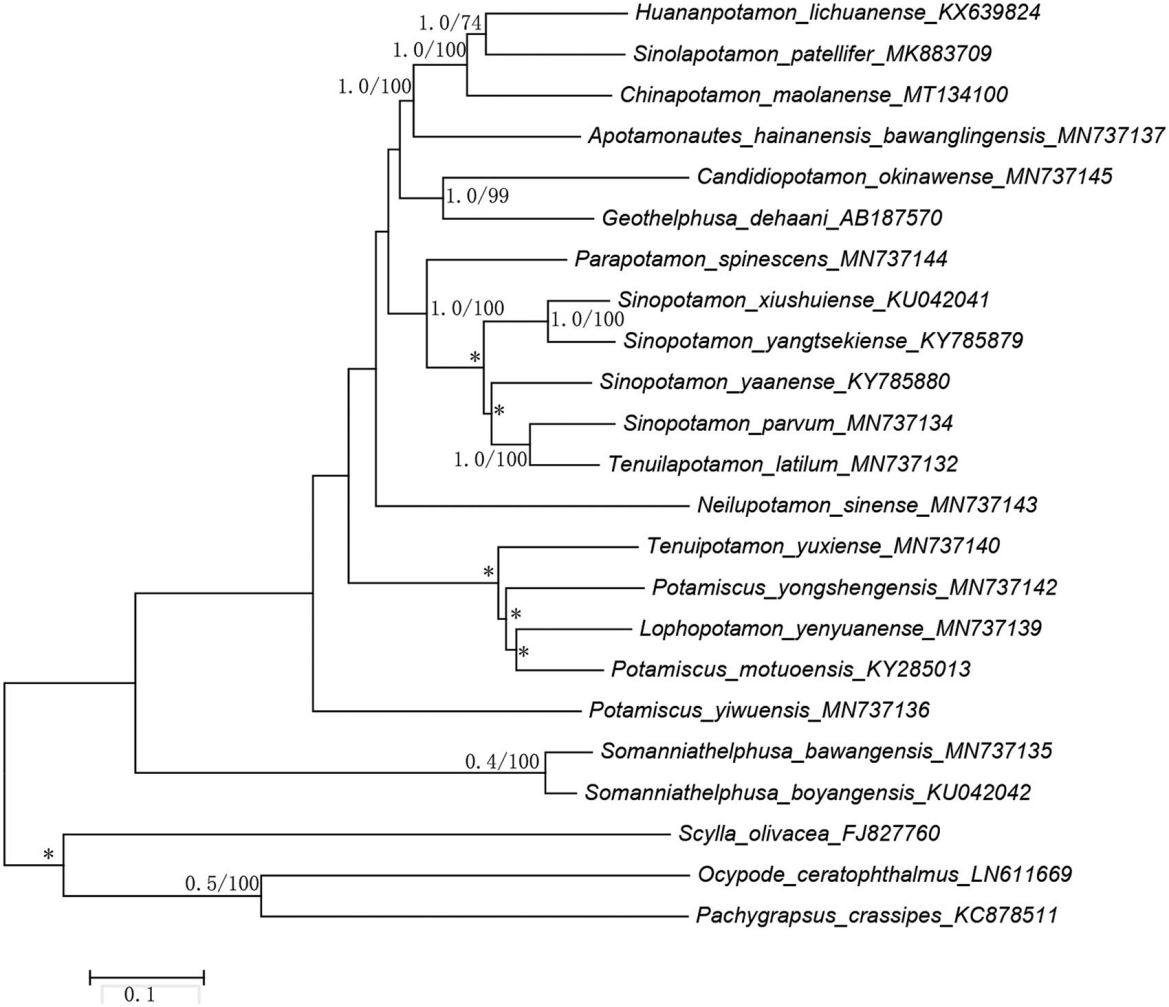
Bayesian Inference (BI) phylogenetic tree of *Chinapotamon maolanense* and other related freshwater crabs based on 13 PCGs in mitogenomes. Numbers on internodes are BI bootstrap proportions and the ML posterior proportions. The same of phylogenetic trees between ML and BI are indicated by bold branches. The differences of freshwater crabs between the ML and BI trees are indicated by ‘*’.The scale bars represent genetic distance.

## Data Availability

The data that support the findings of this study are openly available in GenBank of NCBI at https://www.ncbi.nlm.nih.gov, reference number MT134100.
